# Genotypic and phenotypic relatedness of *Pseudomonas aeruginosa* isolates among the major cystic fibrosis patient cohort in Italy

**DOI:** 10.1186/s12866-016-0760-1

**Published:** 2016-07-11

**Authors:** Cristina Cigana, Paola Melotti, Rossella Baldan, Elisa Pedretti, Emily Pintani, Patrizia Iansa, Ida De Fino, Flavio Favari, Gabriella Bergamini, Gloria Tridello, Daniela M. Cirillo, Baroukh M. Assael, Alessandra Bragonzi

**Affiliations:** Infections and Cystic Fibrosis Unit, Division of Immunology, Transplantation and Infectious Diseases, IRCCS San Raffaele Scientific Institute, via Olgettina 58, 20132 Milan, Italy; Cystic Fibrosis Center, Azienda Ospedaliera Universitaria Integrata di Verona, piazzale Stefani 1, 37126 Verona, Italy; Emerging Bacterial Pathogens Unit, Division of Immunology, Transplantation and Infectious Diseases, IRCCS San Raffaele Scientific Institute, via Olgettina 58, 20132 Milan, Italy; Microbiology and Virology Unit, Department of Pathology, Azienda Ospedaliera Universitaria Integrata di Verona, piazzale Stefani 1, 37126 Verona, Italy; Department of Pathology and Diagnostics, University of Verona, Strada le Grazie 8, 37134 Verona, Italy

**Keywords:** Cystic fibrosis, *Pseudomonas aeruginosa*, Bacterial genotype, Bacterial phenotype, Bacterial adaptation

## Abstract

**Background:**

*Pseudomonas aeruginosa* is the predominant pathogen associated with the decline of pulmonary function in cystic fibrosis (CF) patients. Both environment-to-host acquisition and patient-to-patient transmission have been described for *P. aeruginosa* infection. Epidemic clones and bacterial phenotypic adaptation to the CF lung have been recognised as independent risk factors for disease progression. So far, there is no established link between genotypic prevalence and phenotypic traits. Here, we look at the major CF patient cohort in Italy to identify shared *P. aeruginosa* clones and associated common phenotypic traits.

**Results:**

A comprehensive analysis of *P. aeruginosa* genotypes to determine the presence of high-risk shared clones and their association to specific phenotypic traits has been performed in a major Italian CF centre. Pulsed-field gel electrophoresis (PFGE) of *P. aeruginosa* isolates from 338 CF subjects identified 43 profiles shared by two or more patients and 214 profiles exclusive to individual patients. There was no evidence of a *P. aeruginosa* outbreak, but four most prevalent pulsotypes were detected. Common phenotypic traits were recorded intra-pulsotypes, but we detected heterogeneity inter-pulsotypes. Two of the four major pulsotypes included *P. aeruginosa* isolates with hallmarks of adaptation to the CF airways, including loss of motility, low production of siderophore, pyocyanin and proteases, and antibiotic resistance. One of these pulsotypes grouped a high percentage of hypermutable isolates. No clear correlation between epidemiological and clinical data was found.

**Conclusions:**

We conclude that CF patients of this cohort shared common pulsotypes, but their phenotypic heterogeneity indicates an absence of specific traits associated to *P. aeruginosa* genotypic prevalence.

**Electronic supplementary material:**

The online version of this article (doi:10.1186/s12866-016-0760-1) contains supplementary material, which is available to authorized users.

## Background

*Pseudomonas aeruginosa* is the most common respiratory pathogen in patients with cystic fibrosis (CF) infecting approximately 80 % of subjects, starting from adolescence [1]. The predominant mechanism by which *P. aeruginosa* is acquired is controversial. Few dominant clones, including *P. aeruginosa* PA14 and clone C strain, are distributed worldwide and highly prone to infect CF patients, suggesting environment-to-host acquisition [2, 3]. However patient-to-patient transmission of *P. aeruginosa* has been increasingly reported in a few CF centres [4]. So far, few strains, such as clone C and the Liverpool epidemic strain (LES), have been indicated as highly pathogenic and transmissible causing epidemics within and between several CF clinics [5–9]. LES and the Melbourne strains have also been associated with a worse prognosis and higher rates of mortality, respectively [10, 11]. Thus, person-to-person *P. aeruginosa* transmission may represent a serious threat for CF patients, and this has opened a debate on infection control issues and the management of CF patients.

The pathogenicity of *P. aeruginosa* in CF is promoted by the diversification of the bacterial population and the presence of multiple phenotypes [12]. Common phenotypic traits, such as mucoidy, immotility, type-III secretion system deficiency, *lasR* mutation, hypermutability and lipopolysaccharide (LPS) modifications are consistently acquired by most *P. aeruginosa* strains to promote long-term persistence in CF patients. Few of these *P. aeruginosa* phenotypes (e.g. mucoidy, *lasR* mutant phenotype and hypermutability) have been associated with the more severe lung function [13–15]. While it is well-established that the bacterial intensive genetic adaptation has a key role in the progression of chronic lung infection, the link between specific *P. aeruginosa* phenotypic traits and genotypic prevalence remains to be established.

In this study we addressed a comprehensive analysis of *P. aeruginosa* genotypes at the CF centre in Verona, Italy, to establish the presence of a prevalent clone due to possible patient-to-patient transmission and its association to specific phenotypic traits. Results did not point to the presence of a *P. aeruginosa* outbreak, though sporadic events of possible transmission may have occurred. However, we detected prevalent pulsotypes which are characterised by phenotypic heterogeneity. These data indicate the absence of specific traits in *P. aeruginosa* isolates among prevalent pulsotypes.

## Methods

### Patients and bacterial strains

Between July 2008 and April 2009, 1,352 clinical isolates of *P. aeruginosa* were sampled from 338 patients with CF attending the CF centre in Verona. Patients were followed prospectively and only those intermittently or chronically colonised were selected for the study. Isolation and identification of *P. aeruginosa* from sputum were carried out by plating onto MacConkey agar and incubating for 48–72 h, and by API system 20NE (bioMerieux SA, Lyon, France). Provisional isolate differentiation was made on the basis of colony size, morphology, pigmentation (visual assessment), and mucoidy. *P. aeruginosa* isolates were stored at −80 °C in the MAST CRYOBANK™ (Mast Diagnostics, Bootle, United Kingdom). In the CF centre, patients undergo routine sputum culture four times a year. One positive culture for each CF patient was selected for this study. Four *P. aeruginosa* isolates were collected from this positive culture, and one was blindly chosen for detailed analysis.

### Isolates genotyping

Pulsed Field Gel Electrophoresis (PFGE) of *P. aeruginosa* isolates was performed using the Genepath System apparatus and the CHEF Bacterial Genomic DNA Plug kit (Bio-Rad Laboratories, Hercules, USA), following the protocol by Grundmann et al. [16]. DNA band patterns were analysed with InfoQuest FP software version 5.1 (Bio-Rad Laboratories, Hercules, USA), using the Dice correlation coefficient and Unweighted Pair Group Method with Arithmetic Mean (position tolerance and optimisation of 1.5 %) [17]. By applying the criteria of Tenover *and colleagues* [17], based on the differences in the numbers of bands, we identified a cut-off value of 90 % similarity to correctly cluster the PFGE profiles. A cluster was consequently defined as a group of isolates showing ≥90 % similarity of the PFGE profile.

### Isolates phenotyping

Swimming and twitching capacities were evaluated as described [18]. Proteases, siderophore and pyocyanin secretion, mutation frequency and *lasR* mutant phenotype were assayed as described in the Additional file 1.

### Minimum Inhibitory Concentrations (MIC) measurement

MICs were determined according to Clinical and Laboratory Standards Institute (CLSI) guidelines [19]. MIC_90_ is the concentration active against 90 % of microorganisms tested. Isolates were defined as multidrug-resistant (MDR) and extensively drug-resistant (XDR) according to the terminology approved by the European Centre for Disease Prevention and Control (ECDC) and the Centres for Disease Control and Prevention (CDC) [20].

## Results

### Patients with CF

The clinical data of CF patients selected for the study, including FEV_1_ (Forced Expiratory Volume in 1 s) and their F508del mutation status, are shown in Additional file 1: Table S1. Of the 338 patients, 161 were males and 177 females. The mean age at enrollment was 26.7 years (range 1–50 years). Nineteen pairs of siblings, including four pairs of twins, were included in the study. Seventy-eight patients were homozygous and 147 heterozygous for F508del. The mean FEV_1_ at the time of patient enrollment was 61.5 (range 17–126). The percentage of patients chronically colonised (at least three positive cultures recorded with at least one month intervals between them) by *P. aeruginosa* was 50 %, while the rest were intermittently colonised (with only one or two positive cultures), according to the European Consensus [21].

### Genomic profile of *P. aeruginosa* isolates

**Fig. 1:**
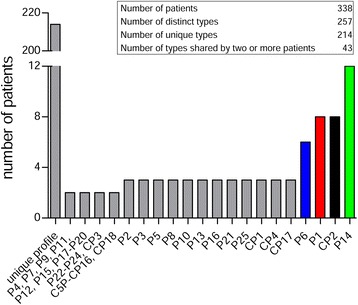
Distribution of isolates among profiles of different sizes. *P. aeruginosa* isolates were subjected to PFGE and DNA band patterns were analysed. The distribution of isolates among the profiles is shown

**Fig. 2:**
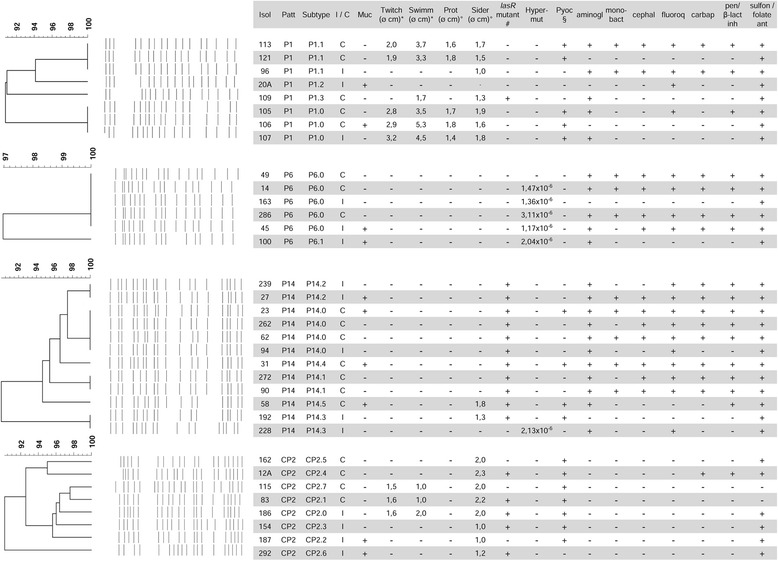
Genotypic and phenotypic characterisation of *P. aeruginosa* isolates belonging to the four more frequent PFGE profiles. PFGE dendrograms of the four major clusters (P1, P6, P14, CP2) are reported. Phenotypic traits of adaptation to CF airways and intermittent or chronic status of isolates are indicated. Resistance to at least one member of a specific antibiotic class is indicated as +. * swimming and twitching motility zone diameter, as measured by subsurface stab assay; ° halo diameter; ^#^ isolates with iridescent and metallic sheen of the colony surface, that is typical for a *lasR* mutant, are indicated as +; ^§^ visible pyocyanin presence +; no visible pyocyanin -; *Abbreviations*: *Isol* isolate, *Patt* PFGE pattern, *I/C* intermittent or chronic, *Muc* mucoidy, *Twitch* twitching motility, *Swimm* swimming motility, *Prot* proteases, *Sider* siderophores, *Hyper-mut* hyper-mutability (mutation frequency), *Pyoc* pyocyanin, *aminogl* aminoglycosides, *mono-bact* mono-bactams, *cephal* cephalosporins, *fluoroq* fluoroquinolones, *carbap* carbapenems, *pen/β-lact inh* penicillins/β-lactamase inhibitors, *sulfon/folate ant* sulfonamides/folate antagonists

PFGE was carried out for all 338 *P. aeruginosa* isolates. By setting the similarity cut-off at 90 %, 214 PFGE profiles were classified as unique pulsotypes of single patients (Fig. 1). Of the 43 clusters of strains detected, 25 clusters included at least two or more *P. aeruginosa* isolates showing identical PFGE profile (100 % similarity) or belonging to highly related subtypes (≥90 % similarity), and these were classified as “PFGE patterns” (P). The remaining 18 clusters grouped two or more *P. aeruginosa* isolates which were highly related to each other (≥90 % similarity), but none of them identical, and these were classified as “PFGE correlated profiles” (CP). Four major PFGE clusters, including more than three isolates, were detected: P1, P6, P14 and CP2 (Fig. 2). The contacts investigation showed no clear link among patients with the same PFGE profile. Cluster P1 included *P. aeruginosa* isolates from eight CF patients, distributed in four highly related subtypes. Two P1 subtypes (P1.0 and P1.1) included three indistinguishable *P. aeruginosa* isolates each. Cluster P6 included isolates from six patients, five of them identical (100 % similarity) (P6.0) and one highly related (97 % similarity) to subtype P6.0. Cluster P14 included isolates from 12 patients, distributed in six highly related subtypes, one of them grouping four indistinguishable isolates (P14.0) and three grouping two indistinguishable isolates each (P14.1, P14.2 and P14.3). Cluster CP2 included isolates highly related to each other derived from eight CF patients’ clustering with a similarity ≥90 % (CP2.0 – CP2.7). The remaining 304 isolates presented unique PFGE profiles (63.3 % of the total strains) or were included in other minor PFGE clusters, grouping two or three *P. aeruginosa* isolates (26.6 %) with a similarity of at least 90 %. One pair of siblings harboured an indistinguishable genotype (P13), while two pairs were infected by highly related *P. aeruginosa* isolates (CP7 and CP18). The genotype and the characteristics of patients with *P. aeruginosa* isolates belonging to the prevalent PFGE clusters (P1, P6, P14 and CP2) are reported in Table 1 and summarized in Table 2. Overall, these findings indicate that a *P. aeruginosa* outbreak was not found, despite the presence of prevalent pulsotypes, potentially due to sporadic cross-transmission.

**Table 1:** Demographics of CF patients harbouring *P. aeruginosa* isolates belonging to the most representative patterns

Isolate	Pattern	Subtype	Sex	Age (yrs range)	FEV_1_ (% predicted)	*P. aeruginosa* status	Age at 1^st^ *P. aeruginosa* isolation (range)
113	P1	P1.1	F	46–50	31	C	26–30
121^a^	P1	P1.1	M	6–10	54	C	0–5
96^b^	P1	P1.1	F	21–25	82	I	16–20
20A	P1	P1.2	F	26–30	68	I	0–5
109^b^	P1	P1.3	M	36–40	52	C	31–35
105	P1	P1.0	M	31–35	66	C	0–5
106	P1	P1.0	M	36–40	70	C	21–25
107^b^	P1	P1.0	M	41–45	84	I	26–30
49^b^	P6	P6.0	M	34	39	C	6–10
14	P6	P6.0	M	36–40	41	C	16–20
163^b^	P6	P6.0	F	16–20	112	I	6–10
286	P6	P6.0	F	21–25	27	C	0–5
45	P6	P6.0	F	26–30	43	I	16–20
100^a^	P6	P6.1	F	31–35	48	I	11–15
239^a^	P14	P14.2	M	21–25	55	I	0–5
27^b^	P14	P14.2	M	21–25	96	I	11–15
23^b^	P14	P14.0	M	26–30	55	C	0–5
262	P14	P14.0	F	31–35	82	C	16–20
62^b^	P14	P14.0	M	31–35	55	C	16–20
94^b^	P14	P14.0	F	31–35	50	I	16–20
31	P14	P14.4	F	31–35	26	C	16–20
272^a^	P14	P14.1	M	11–15	105	C	6–10
90	P14	P14.1	F	21–25	64	C	0–5
58	P14	P14.5	F	16–20	39	C	11–15
192^b^	P14	P14.3	M	26–30	100	I	0–5
228^b^	P14	P14.3	M	31–35	29	I	11–15
162	CP2	CP2.5	M	21–25	61	C	0–5
12A	CP2	CP2.4	F	11–15	51,8	C	0–5
115^b^	CP2	CP2.7	M	36–40	79	C	21–25
83^b^	CP2	CP2.1	M	11–15	100	C	0–5
186^b^	CP2	CP2.0	F	6–10	115	I	6–10
154	CP2	CP2.3	F	31–35	76	I	6–10
187	CP2	CP2.2	F	41–45	50	I	21–25
292	CP2	CP2.6	M	36–40	51	I	21–25

*Abbreviations and symbols*: *M* male, *F* female, *FEV*_*1*_ forced expiratory volume in 1 s, *C* chronic, *I* intermittent; ^a^ F508del +/+; ^b^ F508del +/−

**Table 2:** CF patient demographics

Pattern (N° of patients)	Male %	Mean age (yrs)	FEV_1_ %	F508del homozygous %	Chronically colonised %	Mean age at the first colonisation (yrs)	Annual median rate of decline in FEV_1_ percent predicted over three years (%)
P1 (8)	62.5	32.6 (10–46)	63.4 (31–84)	12.5	75	16.4 (1–32)	4.4 (0.5 to 11.3)
P6 (6)	33.3	27.4 (20–36)	51.7 (27–112)	16.7	67	11.3 (2–20)	2.7 (−9.5 to 12)
P14 (12)	50	26.7 (13–34)	63 (26–100)	14.3	83	10.3 (0–18)	6.1 (−1.5 to 15)
CP2 (8)	50	25.9 (10–41)	73 (50–115)	0	50	11.6 (0–25)	9.2 (−2 to 21.1)
Others (304)	47	26.5 (1–50)	61.3 (17–113)	24.3	67	11.6 (0–47)	5.8 (−37.5 to 39)

### Phenotypic characterisation of the major *P. aeruginosa* clusters

Phenotypic characterisation was carried out on 34 *P. aeruginosa* isolates belonging to the four prevalent clusters (P1, P6, CP2, P14) identified by PFGE analysis (Fig. 2, Additional file 1: Table S2). Most of the isolates in cluster P1 were non-mucoid (75 %), motile (62.5 % with twitching and 75 % with swimming motility), protease, siderophore and pyocyanin producers (respectively 62.5, 87.5 and 62.5 %), non-hypermutable (100 %), *lasR* wt (87.5 %); two isolates were XDR and one isolate was MDR (at least MDR: 37.5 %) [20]. Isolates belonging to cluster P6 were prevalently non-mucoid (66.7 %), non-motile (100 % lacking both twitching and swimming motility), protease and siderophore low-producers (100 % lacking both protease and siderophore secretion), *lasR* wt (100 %), hypermutable (83.3 %) and pyocyanin low producers (100 %). Three isolates were XDR and one was MDR (at least MDR: 66.7 %). Isolates of cluster P14 were mainly non-mucoid (66.7 %), non-motile (100 % lacking both twitching and swimming motility), protease and siderophore low-producers (100 % lacking protease and 83.3 % lacking siderophore secretion), *lasR* mutated (91.7 %), non-hypermutable (91.7 %) and pyocyanin low-producers. Five isolates were XDR and three were MDR (at least MDR: 66.7 %). Cluster CP2 included isolates mainly non-mucoid (75 %), non-motile (62.5 % lacking both twitching and swimming motility), protease non-secreting (100 %), siderophore secreting (100 %), non-hypermutable (100 %), *lasR* mutated (62.5 %); none of the isolates was MDR or XDR. Notably, no clear difference in antimicrobial resistance between isolates from intermittently and chronically colonised/infected patients within the same PFGE profile was observed. Overall, these results demonstrate the phenotypic heterogeneity among major pulsotypes.

**Fig. 3:**
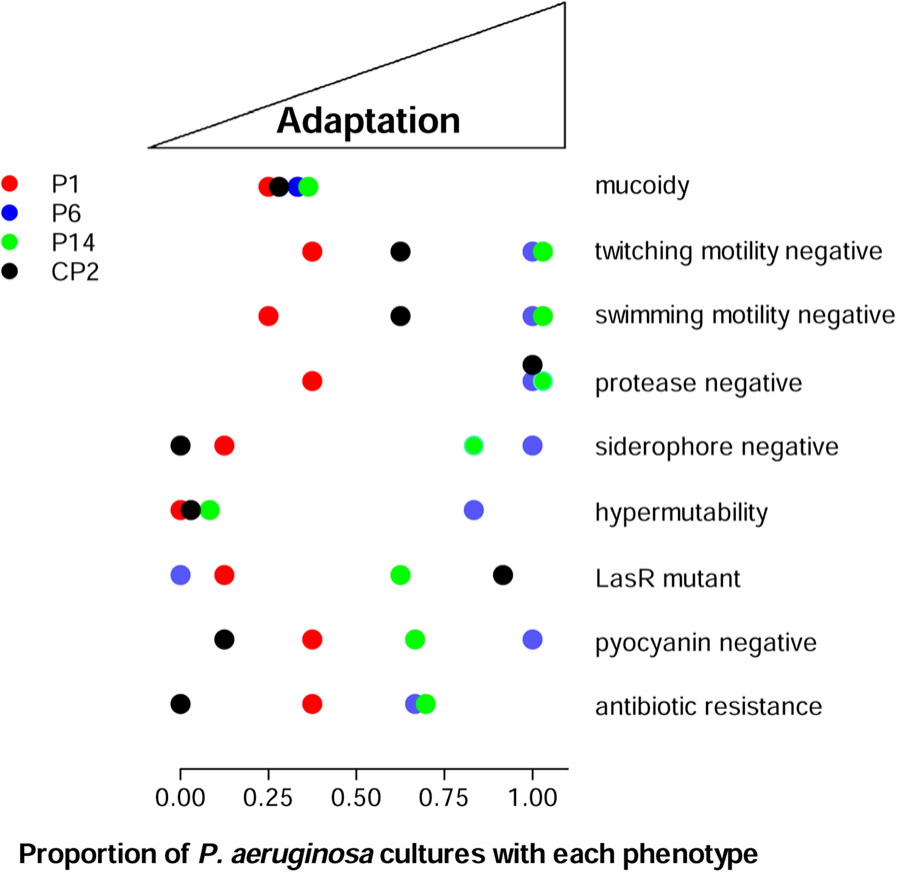
Phenotypic characterisation of *P. aeruginosa* isolates belonging to the four more frequent PFGE profiles. Characterisation of phenotypic traits of adaptation to CF airways were assayed on 34 *P. aeruginosa* isolates belonging to the four major clusters (P1, P6, P14, CP2) identified by PFGE analysis. The proportion of isolates with a specific phenotypic trait was calculated and represented for each profile

Considering all the isolates of the four clusters, it should be pointed out that only a few were mucoid even though several patients were chronically infected. Decrease in the twitching and swimming motility, reduced protease, siderophore and pyocyanin secretion, *lasR* mutant phenotype, hypermutability and MDR phenotype, which are hallmarks of bacterial adaptation, were detected prevalently in cluster P6 (Fig. 3).

## Discussion

This work provides the results of the genotypic characterisation of *P. aeruginosa* isolates collected from 338 patients at the CF centre in Verona between 2008 and 2009. The objective was to establish the presence of a high-risk outbreak due to possible patient-to-patient transmission. The PFGE analysis revealed the presence of 43 clusters that included isolates from two to 12 patients, indicating possible transmission in this clinic. However, a *P. aeruginosa* outbreak was not evident.

In this study the prevalent cluster included 12 subjects representing the 3.6 % of all the CF patients recruited. In other surveys which identified PFGE profiles shared by 7–20 % of patients, the authors concluded that the risk of cross-colonisation was extremely low [22–24]. According to these criteria, at the CF centre in Verona the risk of patient-to-patient transmission is low, although it cannot be excluded in some cases. It has been reported that different management of the *P. aeruginosa* infections in terms of antimicrobial chemotherapy and infection control is probably responsible for the discordant epidemiology [25]. At the CF centre in Verona, strong antibiotic treatment was administered only in the presence of pulmonary exacerbations and for eradication protocols. Overall, the pre-existing rules for infection control in this CF centre and the strategy of eradicating *P. aeruginosa* when isolated for the first time may have counteracted outbreaks of epidemic strains, as shown by our results. Moreover, the structure and organisation of this centre changed in 2012, in accordance with the infection control policies suggested in the European Consensus for the standards of care for CF patients [26]. The implementation of infection control strategies may have further reduced the risk of cross-transmission compared to the study period analysed (2008–2009).

The long-term colonisation of CF patients’ airways by *P. aeruginosa* selects clonal patho-adaptive variants with different features compared to the initially acquired strain [27]. Of these features, the most frequently described are the loss of invasive functions such as twitching and swimming motility or virulence factors as pyocyanin, several proteases, acquisition of mucoid and hypermutable phenotype. These phenotypic changes favour the persistence of *P. aeruginosa* variants in the CF lung. Despite the well-known relevance of *P. aeruginosa* phenotypes, associated to adaptation to the CF airways, to the progression of lung disease, as far as we are aware this is the first work which attempts to examine the link between predominant *P. aeruginosa* pulsotypes and phenotypic traits. Our phenotypic analysis showed that, despite a phenotypic homogeneity within the pulsotype, the clusters were heterogeneous when compared with each other, indicating an absence of specific traits characterising the predominant pulsotypes. More importantly, these data indicate that the potential patient-to-patient transmission cannot be due to a specific *P. aeruginosa* phenotypic trait analysed in this study. However, we cannot exclude that other adaptive traits (e.g., metabolic changes, lipopolysaccharide modifications) may be involved in cross-transmission.

Among the limitations of this study, we acknowledge that environmental microbiologic sampling was not performed. As a consequence we cannot exclude that the most common pulsotypes were acquired from an environmental source. In addition, we only tested a single *P. aeruginosa* isolate per sputum sample. A previous study reported that the great majority of patients only harbour a unique genotype [28]. However, co-infection with multiple *P. aeruginosa* isolates is also well-described in CF patients [29, 30]. Secondly, this study relied on the analysis of isolates recovered from the sputum. And although sputum culture appears to be a reliable means for detecting *P. aeruginosa* in the lower airways [31], it should be considered that different regions of the CF lung may be colonised by different isolates. We cannot exclude that other strains may be dominant in other regions of the CF lung, e.g. the more distal areas which are difficult to access, and probably affected by more stringent environmental conditions (e.g. oxygen depletion). Overall, these two limitations may lead to an underestimation of the prevalent pulsotypes, and of the genetic variability among *P. aeruginosa* isolates. The choice to use PFGE as a typing tool may also be questionable, as it is expensive, labour-intensive and does not allow comparisons with data from other groups, different to the multilocus sequence typing (MSLT) and random amplified polymorphic DNA (RAPD). However, PFGE was considered to be the gold-standard method for microbial typing when the survey was initiated. Recently it was shown that the discriminatory power of PFGE, MSLT and RAPD are comparable [32]. Furthermore, while MSLT was shown to be highly predictive in labelling strains as unique, PFGE was the best technique for predicting clonality. Despite all the criticisms, there is no evidence to support the claim of an outbreak at the CF centre in Verona, although the risk of cross-transmission among patients cannot be excluded.

## Conclusions

This survey suggested an absence of a *P. aeruginosa* outbreak at the CF centre in Verona, despite the presence of prevalent clusters which may suggest cross-transmission in a few of the cases. We suggest that strict segregation procedures for infection control and the strategy of first eradication of *P. aeruginosa* may have reduced the incidence of nosocomial transmission. These surveillance studies, although complex and expensive, have the potential to monitor the emergence of epidemic high-risk clones and implement infection control strategies within the CF centres. In addition, the inter-pulsotypes phenotypic heterogeneity described in this work indicated the absence of specific traits associated to major pulsotypes. Further studies are needed to rule out any contribution of other determinants to transmission.

## Abbreviations

CF, cystic fibrosis; CP, PFGE correlated profiles; FEV_1_, forced expiratory volume in 1 second; MDR, multidrug-resistant; MIC, minimum inhibitory concentrations; MSLT, multilocus sequence typing; P, PFGE patterns; PFGE, pulsed-field gel electrophoresis; RAPD, random amplified polymorphic DNA; XDR, extensively drug-resistant

## Additional file

Additional file 1: Additional Methods and References; **Tables S1.** Demographics of CF patients enrolled in the study; **Table S2.** Evaluation of pyocyanin production by *P. aeruginosa* isolates from CF patients. (DOCX 102 kb)
