# Supplementary material for: Genotypic and phenotypic relatedness of Pseudomonas aeruginosa isolates among the major cystic fibrosis patient cohort in Italy
Source: BMC Microbiol. 2016 Jul 11;16:142. doi: 10.1186/s12866-016-0760-1 (PMC4940697; doi:10.1186/s12866-016-0760-1)
Supplement: Additional file 1: — Additional Methods and References; Tables S1. Demographics of CF patients enrolled in the study; Table S2. Evaluation of pyocyanin production by P. aeruginosa isolates from CF patients. (DOCX 102 kb) [file 12866_2016_760_MOESM1_ESM.docx]

**Genotypic and phenotypic relatedness of *Pseudomonas aeruginosa* isolates among the major cystic fibrosis patient cohort in Italy**

Cigana C, Melotti P, Baldan R, Pedretti E, Pintani E, Iansa P, De Fino I, Favari F, Bergamini G, Tridello G, Cirillo DM, Assael BM, Bragonzi A

ADDITIONAL FILE

**ADDITIONAL methods**

**Protease secretion**. For this assay, 2 μL of an overnight culture were inoculated onto plates of M9 minimal medium (6.81 g/L Na_2_HPO_4_, 30 g/L KH_2_PO_4_, 0.5 g/L NaCl, 1 g/L NH_4_Cl) containing 0.2 % glucose, 2% skim milk and 1% agar and incubated overnight at 37°C. Being the plate white, protease secretion was indicated by a transparent halo around the colony [[1](#_ENREF_1)]. Metalloproteases are excluded from this study, as this assay is unable to detect them.

**Siderophore secretion.** The green-blue dye chromazurol S (CAS) agar assay was performed according to the protocol modified from Schwyn and Neilands [[2](#_ENREF_2)]. 100 mL of CAS stock solution were prepared by dissolving 60.5 mg CAS in 50 mL of water and mixing with 10 mL of Fe(III)Cl_3_ solution (1 mM FeCl_3_ x 6 H_2_O, 10 mM HCl). 72.9 mg HDTMA dissolved in 40 mL of water were added to give a dark blue solution which was autoclaved at 121°C for 30 min. (10 x) LB medium and (10 x) MM9 solution (10 g/L NH_4_Cl, 3 g/L KH_2_PO_4_, 5 g/L NaCl) were autoclaved separately. Piperazin diethansulfonic acid (PIPES) agar was prepared from 50 mL of (10x) MM9 solution diluted in 250 mL of distilled water. Addition of 15.55 g PIPES was neutralized to pH 6.8 with 50% NaOH. Before autoclaving, the solution was filled up to 425 mL with distilled water and solidified by the addition of 1 % agar. After cooling down the agar solution to 50°C, 15 mL (10x) LB medium, 1 mL glycerol, 1 mL 1 M Na_2_SO_4_, 0.5 mL 0.1 M CaCl_2_ and 50 mL CAS stock solution were added to the PIPES agar under sterile condition and immediately poured into the Petri dishes. 2 μL of an overnight culture were inoculated onto the agar and incubated at 37°C for 24 hours. Siderophore production was indicated by an orange halo.

#### Mutation-frequency measurement.

A single P. aeruginosa colony was grown in 5 ml TSB overnight at 37 °C. Thereafter, aliquots from serial dilutions were plated on TSB–agar plates, 100–200 μl undiluted inoculum was plated in the presence of rifampicin (300 μg ml^−1^) and colonies were counted after 36 h at 37 °C. The mutation frequencies on rifampicin were determined relative to the total count of viable organisms plated according to previously established criteria [[3](#_ENREF_3)].

**Phenotypic analysis for *lasR* mutants**. Colony surface iridescent, metallic sheen, a phenotype that specifically identifies *òasR* mutation, was analysed as described previously [[4](#_ENREF_4), [5](#_ENREF_5)].

**Pyocyanin secretion.** King A medium [[6](#_ENREF_6)] was used to investigate pyocyanin production of the *P. aeruginosa* strains. 40 mL of King A medium (20.0 g/L Tryptone, 3.3 g/L MgCl_2_ x 6 H_2_O, 20.0 g/L KOH, 5.5 mL H_2_SO_4_, 10.0 g/L Glycerol; pH 7.2) were inoculated from overnight culture to an OD_600nm_ of 0.2 and incubated with constant shaking (230 rpm, 37 °C). After 26 hours, culture medium was used for the assessment of pyocyanin production by visual assessment [[7](#_ENREF_7)] and by measuring the absorption of the supernatant at 695 nm after sedimentation of the bacterial culture at 8000 rpm for 10 min [[8](#_ENREF_8)].

**ADDITIONAL REFERENCES**

1. Bragonzi A, Paroni M, Nonis A, Cramer N, Montanari S, Rejman J, Di Serio C, Döring G, Tümmler B: ***Pseudomonas aeruginosa* microevolution during cystic fibrosis lung infection establishes clones with adapted virulence**. *Am J Respir Crit Care Med* 2009, **180**(2):138-145.

2. Schwyn B, Neilands JB: **Universal chemical assay for the detection and determination of siderophores**. *Anal Biochem* 1987, **160**:47-56.

3. Oliver A, Canton R, Campo P, Baquero F, Blazquez J: **High frequency of hypermutable *Pseudomonas aeruginosa* in cystic fibrosis lung infection**. *Science* 2000, **288**(5469):1251-1254.

4. D'Argenio DA, Wu M, Hoffman LR, Kulasekara HD, Déziel E, Smith EE, Nguyen H, Ernst RK, Larson Freeman TJ, Spencer DH, Brittnacher M, Hayden HS, Selgrade S, Klausen M, Goodlett DR, Burns JL, Ramsey BW, Miller SI: **Growth phenotypes of *Pseudomonas aeruginosa* *lasR* mutants adapted to the airways of cystic fibrosis patients**. *Mol Microbiol* 2007, **64**:512-533.

5. Hoffman LR, Kulasekara HD, Emerson J, Houston LS, Burns JL, Ramsey BW, Miller SI: ***Pseudomonas aeruginosa lasR* mutants are associated with cystic fibrosis lung disease progression**. *J Cyst Fibros* 2009, **8**:66-70.

6. King EO, Ward MK, Raney DE: **Two simple media for the demonstration of pyocianin and fluorescin**. *J Lab Clin Med* 1954, **44**:301.

7. Huston WM, Potter AJ, Jennings MP, Rello J, Hauser AR, McEwan AG: **Survey of ferroxidase expression and siderophore production in clinical isolates of *Pseudomonas aeruginosa***. *J Clin Microbiol* 2004, **42**(6):2806-2809.

8. Bianconi I, Milani A, Cigana C, Paroni M, Levesque RC, Bertoni G, Bragonzi A: **Positive signature-tagged mutagenesis in *Pseudomonas aeruginosa*: tracking patho-adaptive mutations promoting airways chronic infection**. *PLoS Pathog* 2011, **7**(2):e1001270.

**Table S1:** Demographics of CF patients enrolled in the study.

| Patient | Sex | Age  (range) | FEV_1_ (%) | Age at first *P. aeruginosa* isolation (range) | *P. aeruginosa* status |
| --- | --- | --- | --- | --- | --- |
| 157* | M | 31-35 | 26 | 0-5 | I |
| 233** | M | 31-35 | 26 | 16-20 | C |
| 240* | M | 41-45 | 97 | 26-30 | I |
| 13 | M | 36-40 | 33.9 | 16-20 | C |
| 7 | F | 6-10 | 87 | 0-5 | C |
| 125* | F | 6-10 | 62 | 0-5 | C |
| 144** | F | 25-30 | 46 | 21-25 | C |
| 44 | M | 41-45 | 35 | 31-35 | C |
| 304** | F | 31-35 | 67 | 26-30 | C |
| 158 | M | 6-10 | 87 | 0-5 | I |
| 28** | M | 11-15 | 70 | 6-10 | C |
| 66 | M | 16-20 | 93 | 11-15 | I |
| 281 | F | 21-25 | 80 | 21-25 | I |
| 141* | F | 26-30 | 41 | 21-25 | C |
| 26* | F | 26-30 | 42 | 21-25 | C |
| 126 | M | 11-15 | 82 | 0-5 | C |
| 5 | M | 36-40 | 27 | 31-35 | C |
| 45B** | M | 11-15 | 90 | 6-10 | I |
| 143 | M | 36-40 | 62 | 31-35 | I |
| 261* | M | 31-35 | 126 | 26-30 | I |
| 122* | F | 0-5 | 66.3 | 0-5 | C |
| 291* | M | 26-30 | 56 | 21-25 | I |
| 311* | M | 6-10 | 75 | 0-5 | I |
| 58 | F | 16-20 | 39 | 11-15 | C |
| 197* | M | 0-5 | 80 | 0-5 | I |
| 32* | F | 0-5 | 113 | 0-5 | I |
| 252* | F | 46-50 | 59 | 46-50 | I |
| 177* | F | 21-25 | 89 | 21-25 | C |
| 295 | M | 31-35 | 82 | 26-30 | I |
| 94* | F | 31-35 | 50 | 16-20 | I |
| 296* | M | 21-25 | 69 | 11-15 | C |
| 87** | F | 21-25 | 54 | 0-5 | I |
| 137* | F | 26-30 | 67 | 11-15 | C |
| 79** | M | 21-25 | 73 | 0-5 | I |
| 306 | M | 36-40 | 37 | 16-20 | I |
| 171 | M | 21-25 | 31 | 6-10 | I |
| 292 | M | 36-40 | 51 | 21-25 | I |
| 285* | M | 41-45 | 46 | 21-25 | I |
| 118** | F | 36-40 | 69 | 21-25 | I |
| 266* | F | 31-35 | 38 | 11-15 | I |
| 245 | F | 41-45 | 43 | 21-25 | I |
| 123* | M | 16-20 | 43 | 0-5 | C |
| 35 | M | 21-25 | ND | 6-10 | I |
| 234 | F | 41-45 | 43 | 26-30 | C |
| 203** | M | 41-45 | ND | 21-25 | C |
| 88* | M | 21-25 | 83 | 0-5 | C |
| 310** | F | 16-20 | 28 | 0-5 | I |
| 40** | F | 31-35 | 23 | 16-20 | C |
| 29** | F | 36-40 | 25 | 21-25 | C |
| 256* | F | 31-35 | 73 | 16-20 | I |
| 213** | F | 26-30 | 98 | 0-5 | C |
| 221 | M | 16-20 | 98 | 6-10 | I |
| 214 | M | 16-20 | 85 | 0-5 | I |
| 172* | F | 21-25 | 61 | 11-15 | I |
| 138 | M | 36-40 | 51 | 21-25 | C |
| 13°* | M | 16-20 | ND | 6-10 | C |
| 185 | M | 26-30 | 43 | 6-10 | I |
| 23* | M | 26-30 | 55 | 0-5 | C |
| 307* | M | 26-30 | 36 | 6-10 | I |
| 73** | F | 31-35 | 53 | 31-35 | C |
| 98** | M | 26-30 | 94 | 0-5 | I |
| 6°* | F | 31-35 | 75.3 | 26-30 | C |
| 278* | F | 16-20 | 110 | 0-5 | I |
| 294 | F | 16-20 | 72 | 6-10 | I |
| 204* | M | 16-20 | 37 | 11-15 | C |
| 224* | M | 26-30 | 113 | 0-5 | I |
| 112 | F | 36-40 | 108 | 21-25 | C |
| 253* | M | 31-35 | 27 | 0-5 | I |
| 128** | F | 26-30 | 39 | 0-5 | C |
| 124* | M | 26-30 | 74 | 0-5 | I |
| 225** | M | 36-40 | 38 | 21-25 | C |
| 219** | M | 36-40 | 50 | 21-25 | C |
| 113 | F | 46-50 | 31 | 26-30 | C |
| 90 | F | 21-25 | 64 | 0-5 | C |
| 139** | F | 31-35 | 55 | 0-5 | C |
| 52* | F | 16-20 | 92 | 0-5 | C |
| 250 | F | 21-25 | 74 | 11-15 | I |
| 91* | F | 31-35 | 62 | 0-5 | C |
| 84* | F | 31-35 | 39 | 0-5 | C |
| 181 | F | 16-20 | 68 | 0-5 | C |
| 9* | M | 36-40 | 41 | 16-20 | C |
| 235* | F | 36-40 | 39 | 21-25 | C |
| 114* | F | 16-20 | 59 | 0-5 | C |
| 68* | M | 31-35 | 51 | 31-35 | C |
| 74** | M | 36-40 | 30 | 16-20 | C |
| 246** | M | 31-35 | 84 | 21-25 | C |
| 69 | F | 16-20 | 37 | 0-5 | C |
| 129* | F | 36-40 | 52 | 16-20 | I |
| 167* | M | 41-45 | 71 | 21-25 | I |
| 41* | M | 36-40 | 23 | 21-25 | C |
| 75 | F | 36-40 | 32 | 21-25 | C |
| 48 | M | 26-30 | ND | 0-5 | I |
| 196* | M | 26-30 | 72 | 0-5 | C |
| 24 | M | 26-30 | 49 | 0-5 | C |
| 45 | F | 26-30 | 43 | 16-20 | I |
| 39B** | F | 31-35 | 52 | 0-5 | I |
| 199 | M | 31-35 | 50 | 0-5 | C |
| 247* | M | 21-25 | 51 | 0-5 | C |
| 165 | M | 26-30 | 26 | 0-5 | C |
| 37* | F | 36-40 | 53 | 21-25 | C |
| 150** | M | 36-40 | 28 | 16-20 | C |
| 102 | F | 21-25 | 36 | 0-5 | C |
| 205* | F | 16-20 | 78 | 0-5 | C |
| 215* | F | 21-25 | 70 | 0-5 | C |
| 208 | F | 26-30 | 51 | 0-5 | C |
| 222* | F | 36-40 | 63 | 21-25 | C |
| 268* | M | 26-30 | 38 | 0-5 | I |
| 308* | F | 16-20 | 94 | 0-5 | C |
| 31 | F | 31-35 | 26 | 16-20 | C |
| 140* | M | 36-40 | 49 | 21-25 | C |
| 155* | F | 26-30 | 63 | 6-10 | I |
| 62* | M | 31-35 | 55 | 16-20 | C |
| 80 | F | 31-35 | 69 | 0-5 | I |
| 38B* | F | 21-25 | 75 | 0-5 | C |
| 20 | F | 26-30 | 68 | 0-5 | I |
| 271 | F | 31-35 | 55 | 0-5 | C |
| 189** | M | 26-30 | 60 | 0-5 | I |
| 228* | M | 31-35 | 29 | 11-15 | I |
| 162 | M | 21-25 | 61 | 0-5 | C |
| 298* | M | 26-30 | 22 | 0-5 | I |
| 299** | F | 21-25 | 69 | 0-5 | I |
| 216 | M | 36-40 | 41 | 16-20 | C |
| 105 | M | 31-35 | 66 | 0-5 | C |
| 100** | F | 31-35 | 48 | 11-15 | I |
| 262 | F | 31-35 | 82 | 16-20 | C |
| 35B | M | 26-30 | 97 | 0-5 | I |
| 59 | F | 36-40 | 20 | 16-20 | C |
| 25** | M | 26-30 | 95 | 11-15 | I |
| 166* | F | 16-20 | 44 | 0-5 | C |
| 259** | F | 36-40 | 97 | 21-25 | I |
| 242** | M | 31-35 | 73 | 0-5 | I |
| 254* | M | 41-45 | 39 | 26-30 | I |
| 255** | F | 26-30 | 30 | 11-15 | I |
| 243 | M | 16-20 | 92 | 6-10 | C |
| 81* | M | 41-45 | 58 | 21-25 | C |
| 115* | M | 36-40 | 79 | 21-25 | C |
| 273 | F | 21-25 | 20 | 11-15 | C |
| 133 | M | 21-25 | 26 | 6-10 | C |
| 288 | M | 31-35 | 75 | 16-20 | I |
| 70 | F | 36-40 | 35 | 21-25 | C |
| 269 | M | 26-30 | ND | 11-15 | I |
| 190 | M | 36-40 | 37 | 11-15 | I |
| 168 | F | 16-20 | 109 | 0-5 | I |
| 192 | M | 26-30 | 100 | 0-5 | I |
| 42 | F | 36-40 | 54 | 16-20 | C |
| 119 | M | 16-20 | 33 | 0-5 | I |
| 116 | F | 31-35 | 51 | 0-5 | C |
| 206 | M | 16-20 | 54 | 0-5 | C |
| 279 | F | 16-20 | 95 | 0-5 | I |
| 187 | F | 41-45 | 50 | 21-25 | I |
| 136 | M | 36-40 | 41 | 16-20 | I |
| 106 | M | 36-40 | 70 | 21-25 | C |
| 178 | M | 26-30 | 49 | 6-10 | I |
| 85 | M | 26-30 | 63 | 0-5 | I |
| 95 | M | 21-25 | ND | 0-5 | I |
| 8 | M | 36-40 | 33 | 16-20 | C |
| 293 | F | 31-35 | 46 | 11-15 | C |
| 264 | M | 16-20 | 94 | 0-5 | C |
| 60 | M | 21-25 | 38 | 0-5 | I |
| 76 | F | 36-40 | 64 | 16-20 | C |
| 11 | F | 26-30 | 67 | 0-5 | C |
| 27 | M | 21-25 | 96 | 11-15 | I |
| 183 | M | 31-35 | 40 | 11-15 | I |
| 14 | M | 36-40 | 41 | 16-20 | C |
| 61 | F | 26-30 | 29 | 11-15 | C |
| 300 | M | 31-35 | 53 | 0-5 | I |
| 193 | M | 26-30 | 33 | 0-5 | C |
| 101 | F | 26-30 | 46 | 0-5 | C |
| 164 | M | 26-30 | 44 | 0-5 | I |
| 201 | F | 41-45 | 88 | 26-30 | I |
| 19 | F | 41-45 | 30 | 26-30 | I |
| 56 | F | 26-30 | 57 | 0-5 | I |
| 309 | M | 26-30 | 72 | 0-5 | I |
| 145* | F | 36-40 | 47 | 21-25 | C |
| 286 | F | 21-25 | 27 | 0-5 | C |
| 86 | F | 36-40 | 83 | 21-25 | C |
| 20** | F | 21-25 | 76 | 6-10 | I |
| 120* | F | 41-45 | 41 | 26-30 | C |
| 57* | M | 21-25 | 48 | 0-5 | C |
| 152* | F | 31-35 | 50 | 0-5 | C |
| 97* | F | 31-35 | 32 | 0-5 | C |
| 30* | F | 31-35 | 31 | 0-5 | C |
| 99* | F | 31-35 | 37 | 0-5 | C |
| 71* | F | 26-30 | 58 | 6-10 | C |
| 238* | F | 26-30 | 88 | 21-25 | I |
| 239** | M | 21-25 | 55 | 0-5 | I |
| 154 | F | 31-35 | 76 | 6-10 | I |
| 89 | F | 21-25 | 60 | 0-5 | C |
| 182** | F | 21-25 | 84 | 6-10 | I |
| 194* | F | 21-25 | 84 | 0-5 | C |
| 7°* | F | 16-20 | 93.4 | 0-5 | I |
| 107* | M | 41-45 | 84 | 21-25 | I |
| 280 | M | 36-40 | 93 | 21-25 | C |
| 110* | F | 36-40 | 70 | 21-25 | I |
| 275** | F | 16-20 | 88 | 0-5 | I |
| 263* | M | 16-20 | 84 | 0-5 | C |
| 241** | M | 36-40 | 71 | 21-25 | C |
| 191* | M | 41-45 | 60 | 26-30 | I |
| 3** | M | 11-15 | 84 | 0-5 | C |
| 55 | F | 11-15 | 48 | 6-10 | C |
| 173** | M | 11-15 | 83 | 0-5 | I |
| 134 | M | 41-45 | 34 | 26-30 | I |
| 180 | M | 21-25 | 64 | 6-10 | C |
| 174* | F | 21-25 | 30 | 0-5 | C |
| 53* | F | 16-20 | 58 | 0-5 | I |
| 220 | M | 11-15 | 49 | 0-5 | I |
| 82 | F | 11-15 | 74 | 0-5 | C |
| 92* | M | 26-30 | 52 | 0-5 | I |
| 289 | M | 11-15 | 97 | 0-5 | I |
| 188* | F | 11-15 | 49 | 6-10 | C |
| 146* | F | 31-35 | 50 | 21-25 | C |
| 230** | M | 11-15 | 92 | 0-5 | I |
| 39** | F | 16-20 | 18 | 0-5 | C |
| 147* | M | 36-40 | 32 | 21-25 | C |
| 11* | F | 11-15 | 74 | 0-5 | C |
| 229* | F | 21-25 | 45 | 11-15 | I |
| 267** | F | 11-15 | 99 | 0-5 | I |
| 274* | F | 41-45 | 55 | 26-30 | I |
| 151 | M | 16-20 | 40 | 0-5 | C |
| 163* | F | 16-20 | 112 | 6-10 | I |
| 38 | F | 11-15 | 47 | 0-5 | C |
| 237* | F | 11-15 | 101 | 0-5 | I |
| 34 | M | 36-40 | 39 | 16-20 | I |
| 313** | F | 26-30 | 44 | 6-10 | I |
| 270* | F | 41-45 | 91 | 26-30 | I |
| 211* | F | 11-15 | 78 | 0-5 | C |
| 317* | M | 11-15 | 109 | 0-5 | I |
| 282* | M | 21-25 | 47 | 6-10 | I |
| 49* | M | 31-35 | 39 | 0-5 | C |
| 260** | M | 21-25 | 49 | 11-15 | I |
| 51B** | F | 11-15 | 36 | 0-5 | C |
| 50* | F | 41-45 | 42 | 26-30 | I |
| 160 | F | 11-15 | 81 | 0-5 | I |
| 142** | F | 11-15 | 89 | 0-5 | C |
| 36* | F | 46-50 | 53 | 36-40 | C |
| 316* | M | 11-15 | 110 | 0-5 | C |
| 175 | F | 41-45 | 44 | 26-30 | I |
| 135* | M | 11-15 | 56 | 0-5 | I |
| 272** | M | 11-15 | 105 | 6-10 | C |
| 12 | F | 11-15 | 51.8 | 0-5 | C |
| 46 | F | 11-15 | ND | 0-5 | C |
| 12** | M | 16-20 | 44 | 11-15 | C |
| 176 | M | 16-20 | 69 | 0-5 | I |
| 83* | M | 11-15 | 100 | 0-5 | C |
| 130* | F | 11-15 | 91 | 0-5 | I |
| 283** | F | 16-20 | 108 | 11-15 | C |
| 36B* | M | 11-15 | 100 | 11-15 | I |
| 22A | M | 11-15 | 71 | 0-5 | I |
| 51 | F | 21-25 | 93 | 11-15 | I |
| 16** | M | 16-20 | 34 | 6-10 | C |
| 223 | M | 36-40 | 55 | 21-25 | I |
| 77* | F | 11-15 | 85 | 6-10 | C |
| 17** | M | 16-20 | 100 | 0-5 | C |
| 18** | F | 26-30 | 59 | 0-5 | C |
| 217 | F | 26-30 | 76 | 11-15 | I |
| 93 | F | 31-35 | 91 | 21-25 | C |
| 314 | M | 36-40 | 82 | 26-30 | I |
| 227* | F | 31-35 | 67 | 21-25 | I |
| 127 | M | 16-20 | 63 | 0-5 | I |
| 184* | F | 11-15 | 67 | 0-5 | I |
| 248** | F | 11-15 | 96 | 0-5 | I |
| 170* | M | 11-15 | 106 | 0-5 | C |
| 1 | F | 31-35 | 60 | 11-15 | I |
| 209** | M | 46-50 | 29 | 31-35 | C |
| 63 | M | 31-35 | 24 | 0-5 | C |
| 207* | M | 36-40 | 69 | 16-20 | C |
| 6 | M | 21-25 | 65 | 0-5 | I |
| 121** | M | 6-10 | 54 | 0-5 | C |
| 186* | F | 6-10 | 115 | 6-10 | I |
| 10* | F | 16-20 | 95 | 0-5 | C |
| 265* | F | 6-10 | 58 | 0-5 | I |
| 161** | M | 6-10 | 87 | 0-5 | C |
| 251** | M | 31-35 | 57 | 16-20 | I |
| 284 | M | 31-35 | 55 | 0-5 | I |
| 276 | F | 21-25 | 46 | 11-15 | I |
| 28** | F | 6-10 | 73.3 | 0-5 | C |
| 2* | F | 6-10 | 40.2 | 0-5 | I |
| 21* | F | 6-10 | 55.6 | 0-5 | I |
| 318* | M | 21-25 | 81 | 11-15 | I |
| 78* | M | 21-25 | 30 | 11-15 | C |
| 257** | F | 31-35 | 93 | 16-20 | I |
| 96* | F | 21-25 | 82 | 16-20 | I |
| 27A | F | 6-10 | 72 | 0-5 | I |
| 290** | M | 6-10 | 78 | 0-5 | I |
| 109* | M | 36-40 | 52 | 31-35 | C |
| 26 | F | 31-35 | 64.3 | 21-25 | C |
| 320** | F | 6-10 | 120 | 0-5 | I |
| 132** | F | 6-10 | 82 | 0-5 | I |
| 319 | F | 46-50 | 65 | 46-50 | I |
| 104* | F | 6-10 | 85 | 0-5 | C |
| 64* | M | 41-45 | 60 | 31-35 | I |
| 312 | M | 6-10 | 92 | 0-5 | I |
| 65 | F | 16-20 | 53 | 11-15 | C |
| 1* | M | 6-10 | 64.8 | 6-10 | I |
| 117* | M | 6-10 | 87 | 0-5 | C |
| 218** | F | 16-20 | 87 | 6-10 | I |
| 301 | M | 6-10 | 44 | 0-5 | I |
| 16°* | F | 21-25 | 82.1 | 0-5 | I |
| 287* | F | 36-40 | 52 | 21-25 | I |
| 47** | M | 21-25 | 73 | 0-5 | I |
| 210* | M | 26-30 | 101 | 0-5 | I |
| 43* | M | 31-35 | 65 | 11-15 | I |
| 195** | M | 16-20 | 88 | 0-5 | I |
| 302* | F | 16-20 | 59 | 0-5 | C |
| 249* | F | 36-40 | 63 | 21-25 | C |
| 15* | F | 36-40 | 95 | 21-25 | C |
| 108 | F | 41-45 | 25 | 26-30 | C |
| 17* | F | 16-20 | 43.1 | 0-5 | C |
| 72* | F | 36-40 | 36 | 21-25 | C |
| 303 | M | 26-30 | 91 | 0-5 | I |
| 67** | F | 36-40 | 34 | 21-25 | C |
| 149* | F | 21-25 | 91 | 0-5 | C |
| 202* | M | 26-30 | 18 | 0-5 | I |
| 212* | M | 26-30 | 33 | 0-5 | C |
| 19* | F | 16-20 | 60.3 | 6-10 | C |
| 258 | M | 16-20 | 35 | 0-5 | C |
| 4* | F | 36-40 | 59 | 21-25 | I |
| 179 | F | 36-40 | 50 | 21-25 | C |
| 2 | M | 16-20 | 59 | 0-5 | C |
| 277 | F | 31-35 | 32 | 16-20 | I |
| 54* | F | 31-35 | 60 | 16-20 | I |
| 232 | M | 41-45 | 51 | 21-25 | C |
| 236** | F | 36-40 | 37 | 16-20 | I |
| 103 | F | 31-35 | 97 | 0-5 | I |
| 22** | M | 36-40 | 24 | 21-25 | I |
| 226* | F | 16-20 | 52 | 0-5 | I |
| 9A | F | 0-5 | ND | 0-5 | I |
| 153 | M | 31-35 | 43 | 26-30 | C |
| 33 | F | 36-40 | 49 | 31-35 | I |
| 198* | M | 41-45 | 17 | 41-45 | C |
| 200 | M | 0-5 | ND | 0-5 | I |
| 131 | F | 6-10 | 39 | 6-10 | I |
| 169 | M | 31-35 | 90 | 31-35 | I |
| 231 | F | 26-30 | 54 | 21-25 | I |
| 244* | F | 36-40 | 25 | 36-40 | C |
| 5** | F | 6-10 | 86.2 | 6-10 | I |
| 111 | M | 0-5 | ND | 0-5 | I |
| 159 | F | 36-40 | 43 | 31-35 | C |

** F508del +/+

* F508del +/-

Abbreviations: C, chronical colonisation; I, intermittent colonisation; ND, not determined.

**Table S2.** Evaluation of pyocyanin production by *P. aeruginosa* isolates from CF patients.

| Isolate | Pattern | Subtype | Pyocyanin ^§^ |
| --- | --- | --- | --- |
|  |  |  |  |
| 113 | P1 | P1.1 | 0.11 |
| 121 | P1 | P1.1 | 0.09 |
| 96 | P1 | P1.1 | 0.03 |
| 20A | P1 | P1.2 | 0.03 |
| 109 | P1 | P1.3 | 0.03 |
| 105 | P1 | P1.0 | 0.09 |
| 106 | P1 | P1.0 | 0.1 |
| 107 | P1 | P1.0 | 0.11 |
|  |  |  |  |
| 49 | P6 | P6.0 | 0.02 |
| 14 | P6 | P6.0 | 0.02 |
| 163 | P6 | P6.0 | 0.01 |
| 286 | P6 | P6.0 | 0.02 |
| 45 | P6 | P6.0 | 0.03 |
| 100 | P6 | P6.1 | 0.04 |
|  |  |  |  |
| 239 | P14 | P14.2 | 0.02 |
| 27 | P14 | P14.2 | 0.03 |
| 23 | P14 | P14.0 | 0.12 |
| 262 | P14 | P14.0 | 0.01 |
| 62 | P14 | P14.0 | 0.01 |
| 94 | P14 | P14.0 | 0.02 |
| 31 | P14 | P14.4 | 0.09 |
| 272 | P14 | P14.1 | 0.01 |
| 90 | P14 | P14.1 | 0.01 |
| 58 | P14 | P14.5 | 0.13 |
| 192 | P14 | P14.3 | 0.13 |
| 228 | P14 | P14.3 | 0.02 |
|  |  |  |  |
| 162 | CP2 | CP2.5 | 0.07 |
| 12A | CP2 | CP2.4 | 0.43 |
| 115 | CP2 | CP2.7 | 0.14 |
| 83 | CP2 | CP2.1 | 0.19 |
| 186 | CP2 | CP2.0 | 0.36 |
| 154 | CP2 | CP2.3 | 0.47 |
| 187 | CP2 | CP2.2 | 0.17 |
| 292 | CP2 | CP2.6 | 0.03 |

^§^ OD_695_ at 26h.
